# Joint Inflammation Correlates with Joint GPR30 Expression in Males and Hippocampal GPR30 Expression in Females in a Rat Model of Rheumatoid Arthritis

**DOI:** 10.3390/ijms25147864

**Published:** 2024-07-18

**Authors:** Tanja Grubić Kezele, Hrvoje Omrčen, Lara Batičić, Sandra Šućurović, Sanja Zoričić Cvek

**Affiliations:** 1 Department of Physiology, Immunology and Pathophysiology, Faculty of Medicine, University of Rijeka, 51000 Rijeka, Croatia; 2 Clinical Department for Clinical Microbiology, Clinical Hospital Centre Rijeka, 51000 Rijeka, Croatia; 3 Department of Microbiology, Teaching Institute of Public Health of Primorje-Gorski Kotar County, 51000 Rijeka, Croatia; hrvoje.omrcen@zzjzpgz.hr; 4 Department of Medical Chemistry, Biochemistry and Clinical Chemistry, Faculty of Medicine, University of Rijeka, 51000 Rijeka, Croatia; lara.baticic@uniri.hr; 5 Specialized Hematology Laboratory, Medical Centre Ljubljana, 1000 Ljubljana, Slovenia; sandra.sucurovic@kclj.si; 6 Department of Anatomy, Faculty of Medicine, University of Rijeka, 51000 Rijeka, Croatia; sanja.zoricic@uniri.hr

**Keywords:** cytokines, G protein-coupled estrogen receptor 1, hippocampus, Krenn synovitis score, Mankin osteoarthritis score, microglia, neuroinflammation, rheumatoid arthritis

## Abstract

It is not entirely clear how the interaction between joint inflammation and the central nervous system (CNS) response in rheumatoid arthritis (RA) works, and what pathophysiology underlies the sex differences in coexisting neuropsychiatric comorbidities. It is known that estrogen hormones reduce inflammation in RA and that this occurs mainly via the stimulation of G protein-coupled receptor-30 (GPR30), also known as G protein-coupled estrogen receptor (GPER) 1. However, changes in GPR30 expression and sex differences induced by local and systemic inflammation in RA are not yet known. Our aim was to reveal sex differences in the expression and association of joint GPR30 with local and systemic inflammation, clinical course and furthermore with hippocampal GPR30 expression during pristane-induced arthritis (PIA) in *Dark Agouti* (DA) rats, an animal model of RA. Furthermore, we demonstrated sex-specific differences in the association between joint and systemic inflammation and hippocampal microglia during PIA. Our results suggest sex-specific differences not only in the clinical course and serum levels of pro-inflammatory cytokines but also in the expression of GPR30. Female rats show greater synovial inflammation and greater damage to the articular cartilage compared to males during PIA attack. Male rats express higher levels of synovial and cartilaginous GPR30 than females during PIA, which correlates with a less severe clinical course. The correlation between synovial and cartilaginous GPR30 and joint inflammation scores (Krenn and Mankin) in male rats suggests that the more severe the joint inflammation, the higher the GPR30 expression. At the same time, there is no particular upregulation of hippocampal GPR30 in males. On the other hand, female rats express higher levels of neuroprotective GPR30 in the hippocampus than male rats at the basic level and during PIA attack. In addition, females have a higher number of Iba-1+ cells in the hippocampus during PIA attack that strongly correlates with the clinical score, serum levels of IL-17A, and Krenn and Mankin scores. These results suggest that male rats are better protected from inflammation in the joints and female rats are better protected from the inflammation in the hippocampus during a PIA attack, independently of microglia proliferation. However, in the remission phase, synovial GPR30 expression suddenly increases in female rats, as does hippocampal GPR30 expression in males. Further experiments with a longer remission period are needed to investigate the molecular background of these sex differences, as well as microglia phenotype profiling.

## 1. Introduction

Rheumatoid arthritis (RA) is a chronic autoimmune disease characterized by synovial inflammation and deformation of the joints and adjacent bones, and also involves the development of neuropsychiatric comorbidities [1]. Neuropsychiatric comorbidities in RA contribute to the disease burden and worsen the response to therapy [2,3]. A complex pathological interplay between the local pro-inflammatory mediators (e.g., tumor necrosis factor, TNF; interleukin-17A, IL-17A) and the systemic inflammatory response, including the central nervous system (CNS) inflammatory response, is still being explored. Following the receipt of inflammatory signals from the periphery or the penetration of inflammatory mediators across the blood-brain barrier (BBB), CNS resident cells, particularly microglia, are able to adopt an activated phenotype and maintain a neuroinflammatory state [4]. This state is likely the background of neuropsychiatric symptoms as it causes neuronal damage, impaired neurogenesis in the adult hippocampus, neuronal plasticity, synaptic and network refinement and altered neurotransmitter signaling [5,6]. Importantly, this microglial response in various brain regions can be reversed by inhibiting peripheral human TNF with infliximab, a clinically used agent for the treatment of RA [5]. This in turn explains the link between pro-inflammatory cytokines produced in joint inflammation and activated microglia that drive neuroinflammation in potential psychiatric disorders. Other findings related to experimental arthritis show that microglia can maintain their activated state in brain regions lacking a BBB during persistent autoimmune arthritis, suggesting that chronic inflammation such as RA may affect microglia and have various neural consequences [7]. Furthermore, increased microglial density in the hippocampus contributes to reduced adult neurogenesis and subsequently to neurological sequelae in RA [6,8].

Not only is it not entirely clear how the interaction between joint inflammation and CNS response works, but also what pathophysiology underlies the sex differences in coexisting psychiatric disorders [9]. The reasons why women are generally more affected by RA than men are also not entirely clear, but hormonal aspects are likely to play a role [10,11]. It is known that estrogen hormones reduce inflammation in RA and that this occurs mainly via stimulation of G protein-coupled receptor-30 (GPR30) [12,13,14,15,16,17,18]. GPR30, also known as G protein-coupled estrogen receptor (GPER) 1, is a transmembrane estrogen receptor localized in the plasma membrane and endoplasmic reticulum [13]. It has been shown to exert neuroprotective and anti-inflammatory effects, i.e., GPR30 reduces inflammatory cytokines and BBB permeability, induces neuritogenesis and releases brain-derived neurotrophic factor (BDNF) and regulates synaptic plasticity [14]. It is highly expressed in many brain regions, i.e., in the dentate gyrus (DG), in the CA (*cornu Ammonis*) 1 and CA3 regions [15]. In addition, GPR30 is highly expressed at synapses and is involved in the rapid regulation of dendritic morphology and synaptic plasticity in the hippocampus [16]. In particular, GPR30 is involved in the regulation of anxiety and the development of learning and memory in the hippocampus [17,18]. In addition, GPR30 also mediates cartilage protection in RA [19]. However, there is a lack of information on the expression of GPR30 in synovial and cartilage tissue and its association with that in the hippocampus, a center of adult neurogenesis responsible for neuroregulation and brain health [20]. Improving knowledge of this already complicated network that exists in RA may help to understand the differences between the sexes and elucidate the molecular pathophysiology for new therapeutic approaches.

Accordingly, the aim of this study was to investigate the expression of GPR30 in the hippocampus, synovial tissue and cartilage during different stages of pristane-induced arthritis in *Dark Agouti* (DA) rats, an animal model of RA, and to determine the associations with hippocampal microglia, IL-17A and TNF serum levels, degree of joint inflammation and clinical score in both sexes.

## 2. Results

The clinical course and expression profiles of GPR30 were examined in the hippocampus/DG and in the metatarsophalangeal joints of the hind paws. In addition, the expression of Iba-1+ was examined in the hippocampus/DG. The concentrations of IL-17A and TNF were determined from the blood serum.

### 2.1. Clinical Course

**Figure 1:**
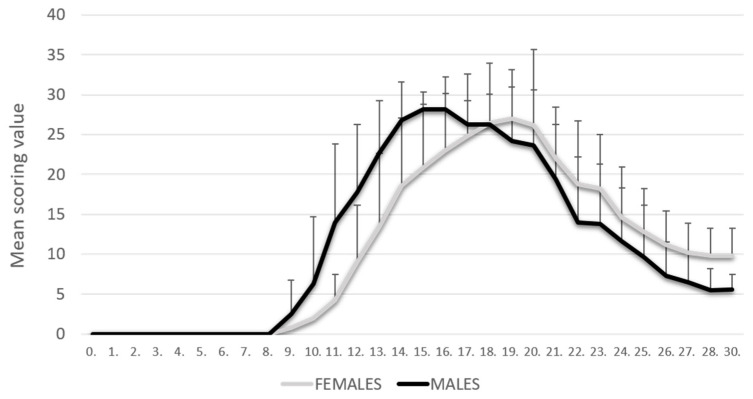
Clinical course. The clinical course in the male (n = 45) and female (n = 45) rat groups. Values are presented as mean ± SD using PIA scores of each animal for every day. Mann–Whitney U test revealed no statistical significance (*p* = 0.847).

The PIA rats were monitored daily for 30 days. As shown in our previous results [8], PIA started earlier in male rats, but they also reached the remission phase earlier. In contrast, female rats had a later onset of disease, but they reached the remission phase with higher clinical scores. There was no statistically significant difference in clinical scores at the peak of the disease (Figure 1).

### 2.2. Female Rats Show Higher Synovial Inflammation and Cartilage Damage than Male Rats

To determine the degree of synovial inflammation, histopathological measurements were performed using the Krenn synovitis scoring system (H&E staining). The data showed an increase in Krenn scores, i.e., low-grade synovitis at the onset of PIA and high-grade synovitis at the peak of PIA in both sexes (Figure S1). However, high-grade inflammation (Krenn score > 5) was present only in female rats in the remission phase of the disease (Figure S1B), with severe proliferation of synovial lining cells (>four layers), high stromal cell density due to persistent inflammatory infiltrate, pannus formation and cartilage and bone destruction. A detailed histological description of the inflamed synovial tissue in both sexes can be found in our previously published results (Figure S1A) [8]. These findings are also consistent with the clinical score (Figure S1C).

The grading of articular cartilage damage during PIA using the modified Mankin scale showed a progressive course of articular cartilage damage (Safranin O/Fast Green FCF). Although the intensity of PIA at baseline and peak was slightly higher in male rats than in females (Figure 1), the damage to articular cartilage was greater in females than in males (Figure S2A,B). The only exception was the onset of PIA, when cartilage damage was significantly more pronounced in males, which is consistent with the clinical course. In remission, a slightly lower degree of articular cartilage damage was observed in both sexes compared to the peak of PIA, but a higher degree in females compared to males, which again corresponds to the clinical course. The degree of articular cartilage damage during PIA did not differ significantly between the sexes, but there was a clear significant difference in both sexes compared to control (*p* < 0.001). These results are also consistent with the clinical score (Figure S2C).

### 2.3. Levels of TNF and IL-17A in Serum

The results showed a statistically significant increase in IL-17A serum levels at disease onset (PIA-onset) in male rats compared to females (*p* < 0.001) (Figure S3A). In addition, the evaluation of TNF serum levels showed a statistically significant increase in the remission phase (PIA-remission) in female rats compared to males (*p* < 0.001) (Figure S3B). These results are consistent with the more progressive onset of PIA in male rats but also with the possibility of the development of chronicity in females (Figure 1). A more detailed description of the correlations between these two pro-inflammatory cytokines and GPR30 expression in synovial tissue, cartilage and hippocampus can be found below.

### 2.4. Expressions and Correlations of GPR30

#### 2.4.1. Male Rats Express a Higher Level of Synovial GPR30 than Female Rats during PIA, but Not in the Remission

**Figure 2:**
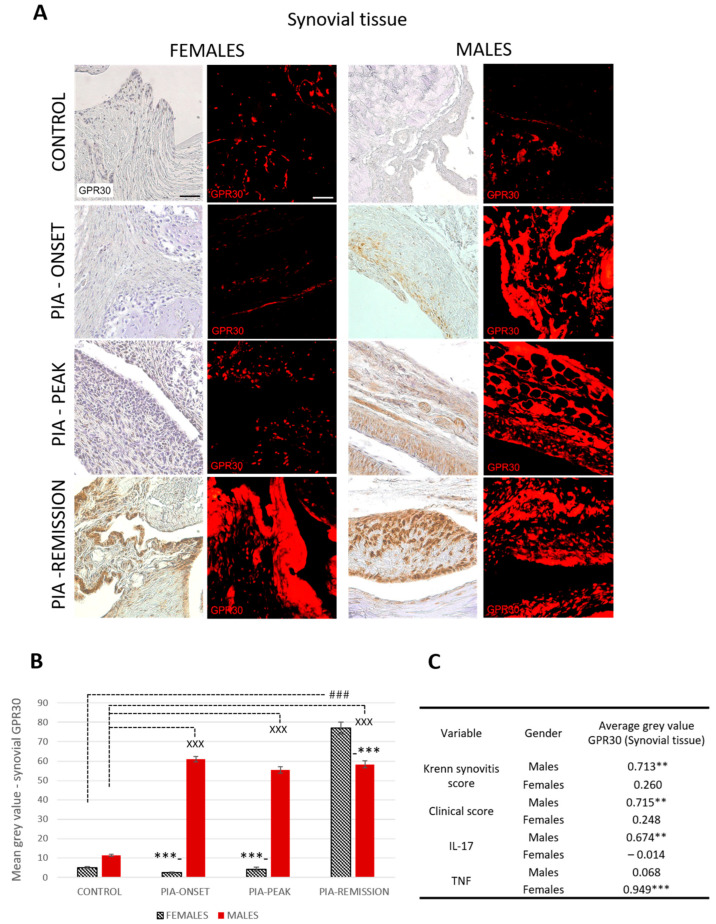
Pristane-induced arthritis upregulates synovial GPR30 expression mostly in male DA rats, which is in strong positive correlation with clinical and Krenn synovitis score. (**A**) Representative immunofluorescent and immunohistological pictures show staining with anti-GPR30 antibodies in paraffin-embedded sections of metatarsophalangeal joints (synovial tissue) obtained from male and female DA rats: control (treated with saline); PIA onset (between 9th and 12th day after induction); PIA peak (between 16th and 20th day after induction); PIA remission (between 20th and 25th day after induction). Scale bars indicate 50 μm. (**B**) Quantification of synovial GPR30 expression was performed using Cell F v3.1 software analysis on 12 regions of interest (3 slides/rat × 6 rats/group = 18 slides/group and total 24 rats). Values are expressed as mean value ± SE. One-way ANOVA followed by the post hoc Scheffé test: * difference between male and female rats; ^X^ difference between male control and male PIA rats; # difference between female control and female PIA rats; ^XXX^ *p* < 0.001; ### *p* < 0.001; *** *p* < 0.001. (**C**) Pearson’s correlation between expression of synovial GPR30 and Krenn synovitis score, clinical score and serum level of IL-17A and TNF: ** *p* < 0.01; *** *p* < 0.001.

Profiling of synovial GPR30 protein in the metatarsophalangeal joints by immunohistochemistry showed significant upregulation in male rats during PIA, starting at disease onset, during the peak phase and remission phase, compared to the results in female rats (Figure 2B: PIA-onset males vs. females, *p* < 0.001; PIA-peak males vs. females, *p* < 0.001; PIA-remission females vs. males, *p* < 0.001). These results are consistent with the clinical course and the increase in serum TNF levels in female rats, especially in the remission phase (Figure S3B), and with the clinical score and serum IL-17A level in males (Figure S3A). Synovial GPR30 expression also correlated with Krenn score in male rats (r = 0.713, *p* < 0.01) (Figure 2C). These results suggest that the more severe the synovial inflammation and the serum level of IL-17A, the higher the synovial GPR30 expression in male rats. On the other hand, the serum TNF level in female rats is strongly correlated with synovial GPR30 expression (r = 0.713, *p* < 0.01) (Figure 2C), implying that the higher the serum level of TNF, the higher the synovial GPR30 expression in female rats.

#### 2.4.2. Male Rats Express a Higher Level of Cartilaginous GPR30 than Female Rats during PIA

**Figure 3:**
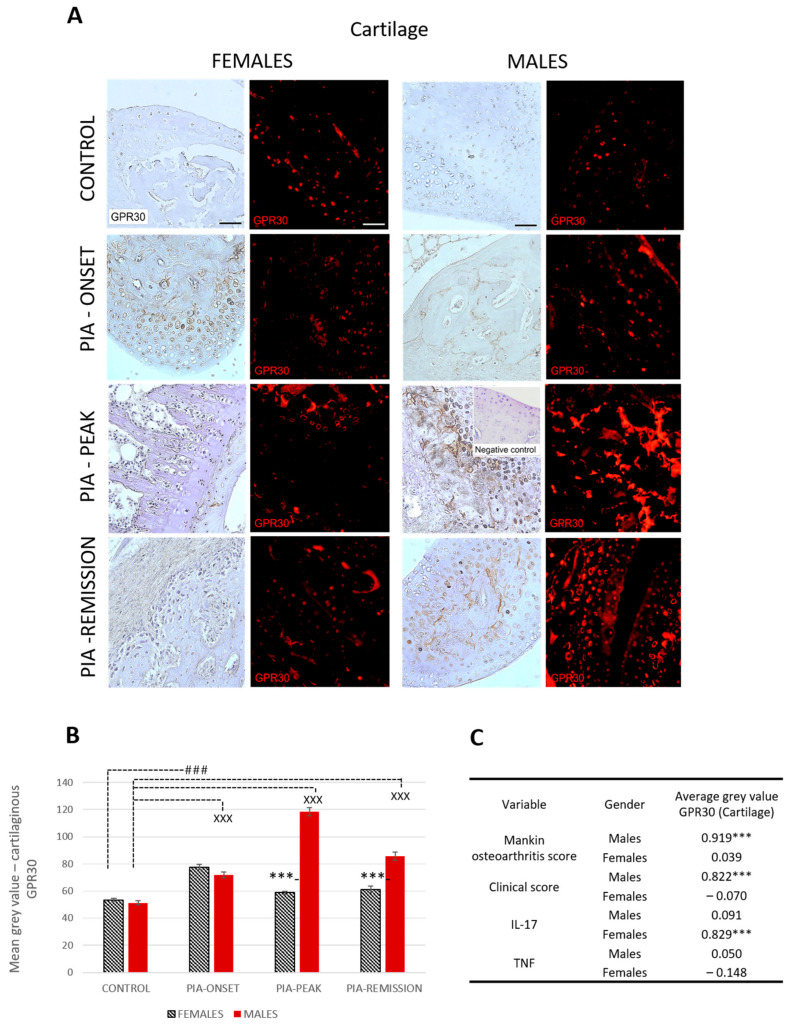
Pristane-induced arthritis upregulates cartilage GPR30 expression mostly in male DA rats, which is in strong positive correlation with clinical and Mankin synovitis score. (**A**) Representative immunofluorescent and immunohistological pictures show staining with anti-GPR30 antibodies in paraffin-embedded sections of metatarsophalangeal joints (cartilage) obtained from male and female DA rats: control (treated with saline); PIA onset (between 9th and 12th day after induction); PIA peak (between 16th and 20th day after induction); PIA remission (between 20th and 25th day after induction). Insert show staining in slides incubated without primary anti-GPR30 antibodies (negative control). Scale bars indicate 50 μm. (**B**) Quantification of cartilaginous GPR30 expression was performed using Cell F v3.1 software analysis on 12 regions of interest (3 slides/rat × 6 rats/group = 18 slides/group and total 24 rats). Values are expressed as mean value ± SE. One-way ANOVA followed by the post hoc Scheffé test: * difference between male and female rats; ^X^ difference between male control and male PIA rats; # difference between female control and female PIA rats; ^XXX^ *p* < 0.001; ### *p* < 0.001; *** *p* < 0.001. (**C**) Pearson’s correlation between expression of cartilage GPR30 and Mankin synovitis score, clinical score, and serum level of IL-17A and TNF: *** *p* < 0.001.

Profiling of cartilaginous GPR30 protein in the metatarsophalangeal joints by immunohistochemistry showed significant upregulation in male rats during PIA, especially during the peak and remission phases, compared to the results in female rats (Figure 3B: PIA-peak males vs. females, *p* < 0.001; PIA-remission females vs. males, *p* < 0.001).

These results are consistent with the clinical course in both sexes (Figure S3A). Cartilaginous GPR30 expression also correlated with the Mankin score in males (r = 0.919, *p* < 0.001) (Figure 3C). This finding implies that the greater the cartilage damage, the greater the cartilaginous GPR30 expression in male rats. On the other hand, the serum IL-17A level in female rats is strongly correlated with cartilaginous GPR30 expression (r = 0.829, *p* < 0.001) (Figure 3C), implying that the higher the serum level of IL-17A, the stronger the cartilaginous GPR30 expression in female rats.

#### 2.4.3. Female Rats Express a Higher Level of Hippocampal GPR30 than Male Rats at Basic Level and during PIA, But Not in the Remission

**Figure 4:**
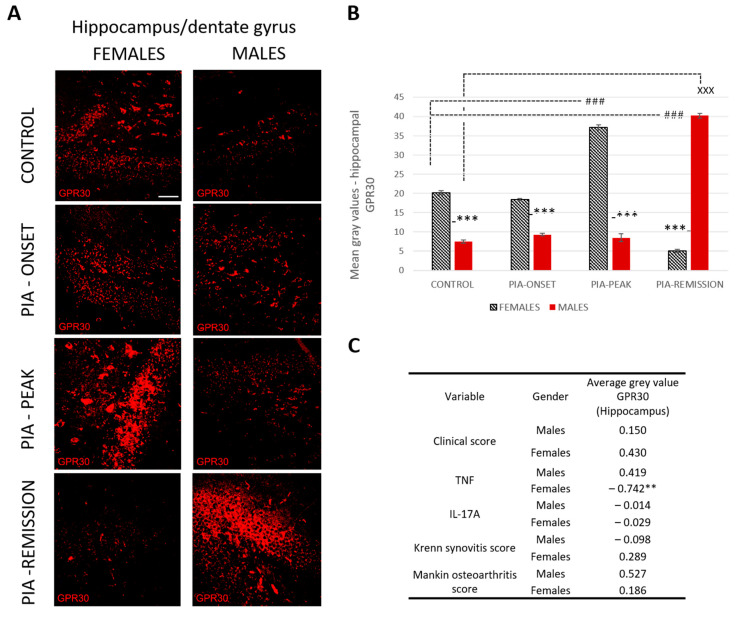
Pristane-induced arthritis upregulates hippocampal GPR30 expression in the acute phase but downregulates in the remission phase of female rats, which is in strong negative correlation with the serum level of TNF. (**A**) Representative immunofluorescent pictures show staining with anti-GPR30 antibodies in paraffin-embedded sections of brain tissue (hippocampus/DG) obtained from male and female DA rats: control (treated with saline); PIA onset (between 9th and 12th day after induction); PIA peak (between 16th and 20th day after induction); PIA remission (between 20th and 25th day after induction). Scale bars indicate 50 μm. (**B**) Quantification of hippocampal GPR30 expression was performed using Cell F v3.1 software analysis on 12 regions of interest (three slides/rat × six rats/group = 18 slides/group and total 24 rats). Values are expressed as mean value ± SE. One-way ANOVA followed by the post hoc Scheffé test: * difference between male and female rats; ^X^ difference between male control and male PIA rats; # difference between female control and female experiment rats; ^XXX^ *p* < 0.001; ### *p* < 0.001; *** *p* < 0.001. (**C**) Pearson’s correlation between hippocampal GPR30 expression, clinical score, serum level of IL-17A and TNF, Krenn synovitis and Mankin osteoarthritis score: ** *p* < 0.01.

Profiling of GPR30 protein in the hippocampus by immunohistochemistry showed significant upregulation in female rats during PIA compared to findings in males (Figure 4B: PIA-onset females vs. males, *p* < 0.001; PIA-peak females vs. males, *p* < 0.001). In the remission phase, however, the female rats showed a significant decrease in GPR30 expression in the hippocampus in contrast to the males (females vs. males, *p* < 0.001). This finding is strongly negatively correlated with the serum level of TNF (r = − 0.742, *p* < 0.01) (Figure 4C and Figure S3B), indicating that the higher the serum level of TNF, the lower the GPR30 expression in the hippocampus in female rats.

### 2.5. Female Rats Express a Higher Number of Iba-1+ Cells in DG of Hippocampus during PIA

**Figure 5:**
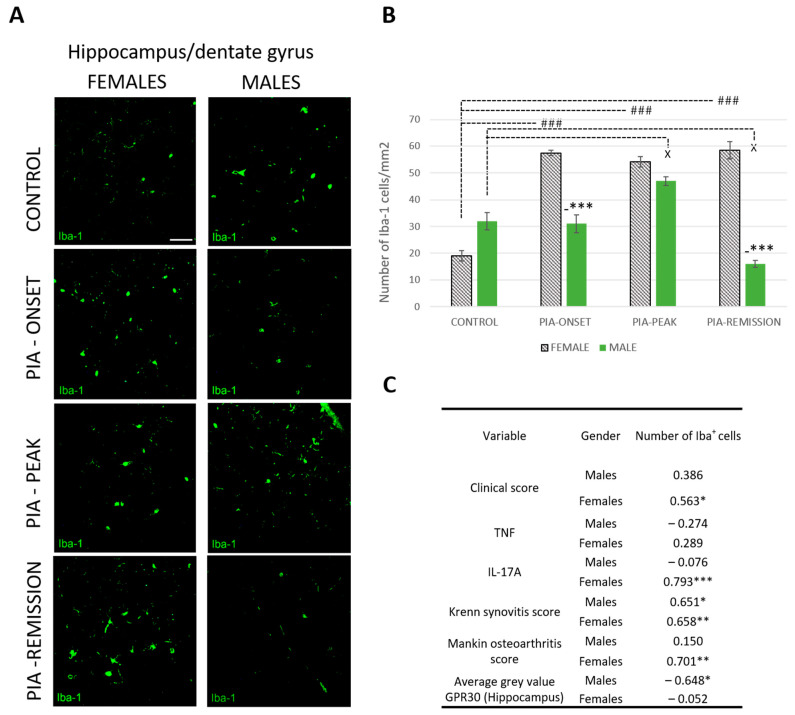
Pristane-induced arthritis upregulates the number of hippocampal Iba-1+ cells mostly in female DA rats, which is in positive correlation with the clinical score, serum level of IL-17A and the Krenn synovitis and Mankin osteoarthritis scores. (**A**) Representative immunofluorescent pictures show staining with anti-Iba-1 antibody in paraffin-embedded sections of brain tissue (hippocampus/DG) obtained from male and female DA rats: control (treated with saline); PIA onset (between 9th and 12th day after induction); PIA peak (between 16th and 20th day after induction); PIA remission (between 20th and 25th day after induction). Scale bars indicate 50 μm. (**B**) The number of hippocampal Iba-1+ cells per mm^2^. Cells were manually counted in regions of interest (12 ROI/4 μm slide x 3 slides/rat × 6 rats/group = 18 slides/group and total 24 rats). Values are expressed as mean value ± SE. One-way ANOVA followed by the post hoc Scheffé test: * difference bewteen male and female rats; ^X^ difference between male control and male experiment rats; # difference between female control and female PIA rats; ^X^ *p* < 0.05, ### *p* < 0.001; and *** *p* < 0.001. (**C**) Pearson’s correlation between the number of hippocampal Iba-1+ cells, hippocampal GPR30 expression, clinical score, serum level of IL-17A and TNF, Krenn synovitis and Mankin osteoathritis score: * *p* < 0.05; ** *p* < 0.01; *** *p* < 0.001.

PIA increased the number of Iba-1+ cells in female rats (Figure 5A,B). This statistically significant difference is most pronounced at the onset of the disease and in the remission phase (*p* < 0.001). These results correlate with the clinical score (r = 563, *p* < 0.05), the serum level of IL-17A (r = 0.793, *p* < 0.001) and both Krenn synovitis (r = 0.658, *p* < 0.01) and Mankin osteoarthritis scores (r = 0.701, *p* < 0.01) in the female rats (Figure 5C), indicating a strong correlation between local and systemic inflammation and the CNS response, i.e., microglial activation in the DG of the hippocampus. Moreover, hippocampal GPR30 expression in male rats showed an inverse correlation with the number of Iba-1+ cells (r = − 0.648, *p* < 0.05), i.e., the higher the hippocampal GRP30 expression, the lower the number of hippocampal Iba-1+ cells, which is not the case in female rats.

## 3. Discussion

In this study, we presented gender-specific differences in the expression of GPR30 in the synovial membrane, cartilage and DG of the hippocampus and their correlation with the clinical score, Krenn synovitis score, Mankin osteoarthritis score, number of Iba-1+ cells in the hippocampus and serum TNF and IL-17A levels.

### 3.1. GPR30 in Joints

There is a lack of information on GPR30 expression and its function in synovial and cartilage tissue in RA. GPR30 regulates chondrocyte proliferation under physiological conditions in female mice and contributes to the longitudinal growth of long bones [21]. In addition, the results of an in vitro study by Sousa et al., 2021, suggest that GPR30 is present in human chondrocytes [22]. They concluded that treatment with GPR30 agonists (17b-estradiol, G-1 and ICI-182780) did not induce Akt or ERK1/2 phosphorylation, suggesting that GPR30 is not functional in human chondrocytes and that the benefit of estrogen-induced responses in human chondrocytes is unlikely to be mediated by GPR30.

However, in the other in vitro study by Fan et al., 2018, who treated cultured ATDC5 chondrocytes with increasing concentrations of 17β-estradiol, the treatment protected these chondrocytes from mitophagy via the GPR30 and PI3K/Akt signaling pathway [23]. Thus, it is likely that the main function of GPR30 in joints is anti-inflammatory and anti-destructive. In addition, the symptoms of cartilage damage and the concentrations of inflammatory cytokines in serum were reduced after estradiol treatment in rats with adjuvant arthritis, in which GPR30 plays an important regulatory role [19]. This implies that chondrocytes express GPR30 under different circumstances and that arthritis could be treated with GPR30 agonists. Furthermore, a natural organic compound, biochanin A, with its anti-inflammatory activity, resolves neutrophilic inflammation in antigen-induced arthritis by acting in the key steps of inflammatory resolution that require the activation of GPR30 and stimulation of cAMP-dependent signaling [24]. All these findings suggest that the actions taken via GPR30 contribute largely to protective actions.

We therefore assume that a higher expression of GPR30 during PIA contributes to better protection against inflammation and destruction during local inflammatory processes in the joint and to faster recovery or remission, as we discovered in male rats in our study. Namely, we found a significant upregulation of GPR30 in male rats during PIA attack (PIA-onset and PIA-peak) and in the remission phase compared to the results in females. These findings are consistent with lower levels of inflammation and cartilage destruction and also correlate strongly with the clinical score in male rats. In addition, we found a correlation between serum levels of IL-17A and synovial GPR30 in male rats. This is consistent with the earlier onset of PIA in males. It is possible that IL-17A is the main trigger for local and systemic inflammatory responses in male rats, including upregulation of local articular GPR30. It has already been confirmed that GPR30 acts as a novel regulator of inflammatory responses, i.e., mediates the reduction of pro-inflammatory cytokines such as TNF and IL-17 and subsequently reduces the severity of the disease [25]. Namely, treatment with the selective GPR30 agonist G-1 partially attenuates TNF-induced upregulation of pro-inflammatory proteins such as intercellular cell adhesion molecule-1 (ICAM-1) and vascular cell adhesion molecule-1 (VCAM-1) [26].

On the other hand, the female rats in our study have more pronounced joint inflammation and cartilage destruction without a significant increase in the expression of GPR30 in synovial tissue during PIA attack. Only articular cartilage showed an upregulation of GPR30 at the onset of PIA. This indicates that GPR30 does not protect the joints of female rats unlike those of males, which can also be confirmed by the more pronounced clinical course in females. This initial increase in cartilaginous GPR30 expression correlates with the slight initial increase in serum IL-17A. However, the only significant increase in synovial GPR30 expression that we found occurs in synovial tissue during the remission phase, when serum levels of TNF significantly increase. This suggests a close positive relationship of serum TNF level with GPR30 expression in the joints of female rats, in contrast to its relationship with GPR30 expression in the hippocampus. However, these results (increase in serum level of TNF) may also indicate a systemic response that further contributes to possible neuroinflammation. Whether this is a late protective response to local joint inflammation and a late upregulation of synovial GPR30 with subsequent downregulation of GPR30 in the CNS should be further clarified.

### 3.2. GPR30 and Microglia in DG/Hippocampus

Activation of GPR30 in the hippocampus has an anxiolytic effect, regulates recognition and aversively motivated memory in male rodents [27,28]. However, in our study, we found no significant increase in GPR30 expression in the hippocampus during PIA attack in male rats, but only in remission, which is in contrast to the results in females.

Indeed, GPR30 expression in the hippocampus is significantly increased in all phases of PIA in females, except in the remission phase. Moreover, our previous results [8] have shown that adult neurogenesis in the hippocampus improves faster in female rats, which may be related to an increased basal expression of the neuroprotective GPR30, and is further enhanced as the disease progresses. This could be because activation of GPR30 increases neurogenesis and reduces neuroinflammation [14]. On the other hand, it could be that this faster improvement is only transient, as we did not measure mature neurons, only immature ones, and a neuroprotective role for GPR30 in mature neurons has already been demonstrated [29]. Furthermore, TNF shows a negative correlation with hippocampal GPR30 expression in female rats, suggesting a negative effect of this pro-inflammatory cytokine on triggering neuroinflammation by downregulating GPR30 expression in the remission. In addition, serum levels of IL-17 correlate positively with hippocampal microglia in female rats, again suggesting that the CNS is more affected by the systemic inflammation of RA than in males. This is supported by the positive correlation between joint inflammation and hippocampal microglia in female rats. However, as we have already said, obviously, this has no impact on adult neurogenesis that progressively improves in remission in females unlike in males [8]. We assume GPR30 upregulation from the beginning could have a greater beneficial impact on hippocampal homeostasis in females. These contradictory findings of simultaneous microglia proliferation and increased GPR30 expression need to be further clarified.

Although the results strongly suggest the introduced link between local joint inflammation and the CNS response in this RA model, the study has more of a correlative rather than a mechanistic approach. Therefore, to confirm the proposed mechanisms underlying this link, further investigation should be conducted using different GPR30 agonists and inhibitors as well as ovariectomized female rats or GPR30 knockout mice. In addition, the profile of the microglia phenotype should be investigated in detail, as we have not analyzed the pro- or anti-inflammatory polarization state of the proliferated microglia. Furthermore, we did not perform memory nor anxiety tasks in this study to confirm the connection between the hippocampal GPR30 upregulation and behavior in both sexes. Although in another RA model (type II collagen-induced arthritis in mice), some mechanisms of arthritis amelioration involve the mediation of nuclear estrogen receptor alpha (ERα) and not GPR30 [30]. Therefore, future investigation should include the analysis of both types of estrogen receptors in pristane-induced arthritis, the transmembrane GPR30 along with the nuclear ones (ERα and ERβ).

Ultimately, some sex differences observed in rats can be extrapolated to RA in humans, taking into account the variability of the reproductive cycle between species and other differences in sex-specific endocrine mechanisms. These include the investigation of synovial and serum levels of certain pro-inflammatory cytokines and estrogen receptors and their correlations with neuropsychiatric comorbidities in both sexes.

## 4. Materials and Methods

### 4.1. Experimental Animals

Experiments were performed on 7–8 week old male and female DA rats. Animals were housed under standard light, temperature and humidity conditions with unlimited access to food and water. Experimental procedures were in compliance with Croatian laws and regulations (OG 135/06; OG 37/13; OG 125/13; OG 055/2013) and with the guidelines set by the European Community Council Directive (86/609/EEC). The experimental protocol was approved by the Croatian Ministry of Agriculture (525-10/0255-19-5; 22 July 2019) and the Ethics Committee of the University of Rijeka, Faculty of Medicine (003-08/21-01/38; 18 June 2021).

### 4.2. Arthritis Induction and Disease Scoring System

**Figure 6:**
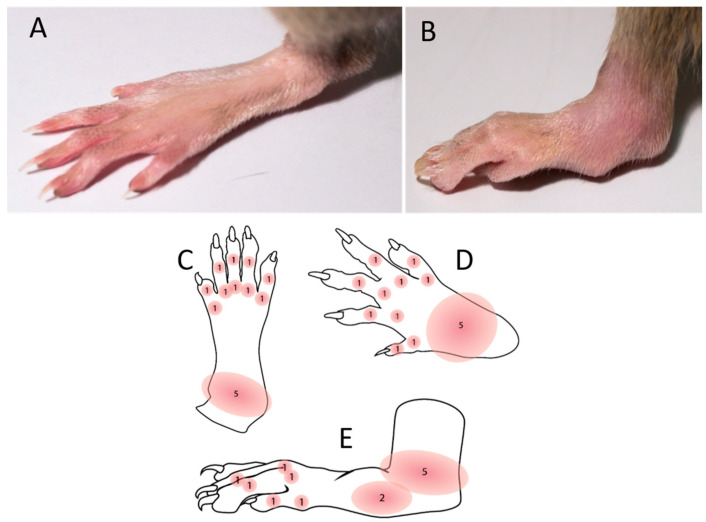
Disease scoring system. The maximum score per limb and rat was 15 and 60, respectively. Scores were not given for deformations if they were not accompanied by erythema or swelling. (**A**) Normal hind paw without the inflammation. (**B**) Inflammed and swolen ankle of a hind paw after PIA induction. (**C**) Illustration of a hind paw from the dorsal side with score points. (**D**) Illustration of a front paw from the dorsal side with score points. (**E**) Illustration of a hind paw from the medial side with score points.

PIA was induced as described in our previously published paper [8]. PIA was induced in genetically susceptible DA rats with an incidence of 99.6% (269 out of 270 animals) in both genders. Arthritis development was monitored and scored in all four limbs using a semi-quantitative scoring system every day after injection [31]. Briefly, one point was given for each swollen or red finger/toe (interphalangeal joints), one point was given for each swollen or red metacarpophalangeal/metatarsophalangeal joint, and five points were given for a swollen wrist/ankle, depending on severity (Figure 6). The day of disease remission is defined here as the first of at least three consecutive scoring days with declining arthritis scores. Control rats were intradermally injected with physiological saline.

### 4.3. Experimental Design

Both female and male rats were randomly divided into two main groups: the control group and the experimental group with induced PIA. PIA rats were further divided into three subgroups sacrificed at different time points after PIA induction, i.e., on the day when the first symptoms appeared between the 9th and 12th day (PIA onset; females: n = 45, males: n = 45), on the peak of disease activity between the 16th and 20th day (PIA peak; females: n = 45, males: n = 45) and in the remission phase between the 20th and 25th day (PIA remission; females: n = 45, males: n = 45). The control group was sacrificed after a few days after saline injection (females: n = 15, males: n = 15). Rats were sacrificed by exsanguination in deep anesthesia induced by a combination of ketamine (80 mg/kg) and xylazine (5 mg/kg) administered intraperitoneally [4,8,32]. All experiments were performed according to the guidance of the European Community Council Directive (86/609/EEC) and recommendation of the National Centre for the Replacement, Refinement and Reduction of Animals in Research.

### 4.4. Tissue Preparation for Paraffin Slices (Brain and Joints)

**Figure 7:**
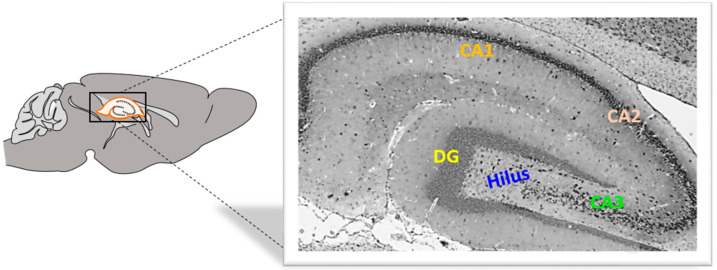
Rat hippocampus from the medial side. DG, dentate gyrus, CA, *cornu Ammonis*. Magnification 40×.

Rat brain hemisphere samples were fixed in 4% buffered paraformaldehyde (Sigma-Aldrich, St. Louis, MO, USA) solution for 24 s. The tissue was then embedded in paraffin wax, and sections were cut at 4 μm using a HM340E microtome (Microtome, Nieder-Olm, Germany) (Figure 7).

Hind paws from rats were collected and fixed in 4% buffered paraformaldehyde (Sigma-Aldrich, St. Louis, MO, USA) solution for 72 h, after which the specimens were immersed in 70% ethanol for 20 min, and the hind paws were then decalcified using a commercial decalcifying solution (Osteofast 2, Biognost, Zagreb, Croatia) for 12 h with the solution being changed every 6 h. The decalcification endpoint was determined by puncturing the specimen with a very thin needle (27 G). Once decalcified, the specimens were washed in three changes of PBS (20 min each), followed by paraffin embedding. Serial paraffin-embedded tissue sections of the hind paws were cut to 4 μm thickness and were incubated for 12 h under 37 °C until the samples were completely dry.

### 4.5. Histological, Immunohistochemical and Immunofluorescence Staining

#### 4.5.1. Histochemistry

For orientation, the rat hind paw slides were stained with hematoxylin and eosin. After deparaffinization in two changes of xylene and rehydration in graded ethanol, slides were stained with Instant Hematoxylin (Thermo Shandon, Pittsburgh, PA, USA) for 5 min, washed under running tap water and then counterstained with Instant Eosin-Y (Thermo Shandon, Pittsburgh, PA, USA) for 10 min. Slides were rinsed in distilled water, dehydrated in graded ethanol and mounted with Entellan (Sigma-Aldrich, St. Louis, MO, USA).

#### 4.5.2. Immunohistochemistry

Immunohistochemical labelling of GPR30 was performed on paraffin-embedded tissues. After deparaffinization and rehydration in graded ethanol, slides were subjected to heat antigen retrieval with citrate buffer (0.01 M Sodium Citrate pH 6.0) for 10 min at 90 °C and then cooled under running tap water. Slices were then incubated with 3% BSA for 15 min to eliminate background staining.

After rinsing in PBS, rabbit monoclonal IgG anti-GPR30 (Abcam, Cambridge, MA, USA, ab260033, diluted 1:100 with 1% BSA in PBS) antibody was added to tissue samples and incubated overnight at 4 °C in a humid environment. The next day, slides were treated with Dako REAL Peroxidase-Blocking Solution (DAKO, Glostrup, Denmark) for 10 min to block endogenous peroxidase activity. Slides were then rinsed in PBS and treated with Streptavidin HRP Conjugate (Invitrogen, Rockford, IL, USA) for 15 min. After rinsing, the slides were incubated with secondary antibodies for 1 h.

The immunoreaction was visualized by adding substrate chromogen (DAB) solution. Slides were counterstained with hematoxylin, dehydrated through graded alcohol, and mounted using Entellan (Sigma-Aldrich, St. Louis, MO, USA). The specificity of staining was confirmed with negative controls. Tissue samples were treated with an identical procedure under the same conditions but with the omission of polyclonal primary antibodies. The photomicrographs were taken and examined under an Olympus BX51 light microscope equipped with an Olympus DP70 camera (Olympus, Tokyo, Japan).

#### 4.5.3. Immunofluorescence

Immunofluorescence labelling was also performed on paraffin-embedded tissue sections. Nonspecific binding was blocked by one-hour incubation with 1% BSA in PBS containing 0.001% NaN3 at room temperature, as previously described [33]. The following primary antibodies were used: rabbit monoclonal IgG anti-GPR30 and goat polyclonal IgG anti-Iba-1 (Abcam, Cambridge, UK, 1:200). Primary antibodies were diluted in blocking solution and incubated with tissue sections overnight at 4 °C in a humid environment. For visualization of immunocomplexes, the following secondary antibodies were used: Alexa Fluor donkey anti-rabbit IgG 594 nm (Molecular Probes, Carlsbad, CA, USA, 1:500) and Alexa Fluor donkey anti-goat IgG 488 nm (Molecular Probes, Carlsbad, CA, USA, 1:300). Secondary antibodies were diluted in blocking solution and incubated with tissue sections in the dark for 1 h at room temperature in a humid environment. Slides were afterwards washed in PBS and mounted with Mowiol (Sigma-Aldrich, Darmstadt, Germany). The photomicrographs were taken under a fluorescent microscope equipped with a DP71CCD camera (Olympus, Tokyo, Japan) and Cell F imaging software. The specificity of the reaction was confirmed by omission of primary antibodies on slides treated with an identical procedure under the same conditions. The photomicrographs were taken and examined under an Olympus BX51 light microscope equipped with an Olympus DP70 camera (Olympus, Tokyo, Japan).

### 4.6. Immunohistochemical/Fluorescence Staining Quantification, Cell Counting and Histopathological Evaluation of Joint Inflammation

#### 4.6.1. Quantification

The immunohistochemical/fluorescence staining quantification of protein expression was performed on 4 μm tissue sections from paraffin-embedded tissues of the brain and hind paws using Cell F v3.1 software (Olympus Soft Imaging Solutions), as previously described [4]. Captured images were subjected to intensity separation. They were sub-sequently inverted, resulting in grayscale images with different intensity ranges, depending on the strength of immunohistochemical staining or fluorescence signals. Regions of interest (ROIs) were arranged to cover the analyzed area, and mean grey values were measured. ROI surface size was always equal for each analyzed area. Twelve ROIs were analyzed per field (400×) on three separate microscopic slides of different tissue samples per animal, obtained from five animals per group. The data were expressed as the mean grey value ± SD.

#### 4.6.2. Cell Counting

We estimated the Iba-1+ cells in the DG using antibodies, and cells were counted manually in an image surface area of 0.053 mm^2^ and at a magnification of 400×. Results were expressed as a mean number of cells per mm^2^ [4].

#### 4.6.3. Histopathological Evaluation of Synovitis

Synovial inflammation was scored with the semi-quantitative Krenn synovitis scoring system, which examines the synovial lining layer cell hyperplasia, stromal cells activation and inflammatory infiltrate using routine hematoxylin and eosin- (H&E) stained slides. All components were graded from 0 to 3, with a maximum score of 9 [34]. Low-grade synovitis was defined by a cumulative score between 2 and 4, while high-grade synovitis was defined by a cumulative score higher than 5.

#### 4.6.4. Histopathological Evaluation of Cartilage Damage

Grading of articular cartilage damage of metacarpophalangeal/metatarsophalangeal joint was scored with the semi-quantitative Mankin osteoarthritis scoring system [35], which examines erosion of articular cartilage, the spatial distribution of chondrocytes and the intensity of staining of the cartilage matrix and chondrocyte territory using Safranin O/Fast Green FCF. The total sum of points according to the Mankin scale indicates the degree of articular cartilage damage, with a higher sum of points indicating greater damage, and ranges from 0 to 15 points.

### 4.7. Determination of Serum Concentrations of IL-17A and TNF

The quantification of interleukin IL-17A and TNF levels in serum in defined rat groups were determined quantitatively using commercially available ELISA kits (Invitro-gen, ThermoFisher Scientific, Waltham, MA, USA). Analytical procedures were performed according to the manufacturer’s instructions. According to the standards, readouts and calculations of concentrations were performed using a microplate reader (Bio-Tek EL808, Winooski, VT, USA) and KC Junior software of Bio-Tek Instruments Inc., KC EL808. Results are expressed as pg/mL of serum (mean ± SD).

### 4.8. Statistical Analysis

The data were evaluated with Statistica, version 13 (TIBCO Software Inc., 2017, Palo Alto, CA, USA). Data distribution was tested for normality using the Kolmogorov–Smirnov test. Differences between genders and groups were assessed with either one-way analysis of variance (ANOVA) followed by the post hoc Scheffé test or Mann-Whitney U test. Pearson correlation (r) was used to determine the associations between GPR30 expression in the hippocampus, synovial tissue and cartilage, serum levels of TNF and IL-17A, Mankin and Krenn clinical scores and Iba-1+ cells in the DG of the hippocampus. The data were expressed as mean ± SD, and the significance level was set at *p* < 0.05.

## 5. Conclusions

Overall, during PIA attack (onset and peak of disease), synovial inflammation with cartilage damage in male rats correlates positively with a greater local (articular) expression of GPR30, and at the same time, there is no particular response or upregulation of GPR30 in the hippocampus. In contrast, in female rats, synovial inflammation and cartilage damage correlate positively with systemic inflammation, i.e., with an increase in pro-inflammatory cytokines, with an additional increase in hippocampal GPR0 expression and the number of Iba-1+ cells. In the remission phase, however, synovial GPR30 expression suddenly increases in female rats, as does hippocampal GPR30 expression in males. Further experiments with a longer remission period are needed to investigate the molecular background of these sex differences. In other words, these results suggest that male rats are better protected from inflammation in the joints and female rats are better protected from the inflammation in the hippocampus during a PIA attack, independently of microglia proliferation. Our data show sex-specific differences in the expression of GPR30 in the joints and hippocampus, which could indicate different protective mechanisms during RA between the sexes and thus a different interplay between the local joint inflammation and the systemic inflammation as well as the accompanying CNS response and possible subsequent neurological consequences.

## Data Availability

Data is contained within the article and Supplementary Materials.

## Supplementary Materials

The following supporting information can be downloaded at: https://www.mdpi.com/article/10.3390/ijms25147864/s1.
