# Supplementary material for: Joint Inflammation Correlates with Joint GPR30 Expression in Males and Hippocampal GPR30 Expression in Females in a Rat Model of Rheumatoid Arthritis
Source: Int J Mol Sci. 2024 Jul 18;25(14):7864. doi: 10.3390/ijms25147864 (PMC11277240; doi:10.3390/ijms25147864)
Supplement: Supplementary file 1 [file ijms-25-07864-s001.zip › ijms-3088680-supplementary.pdf]

A

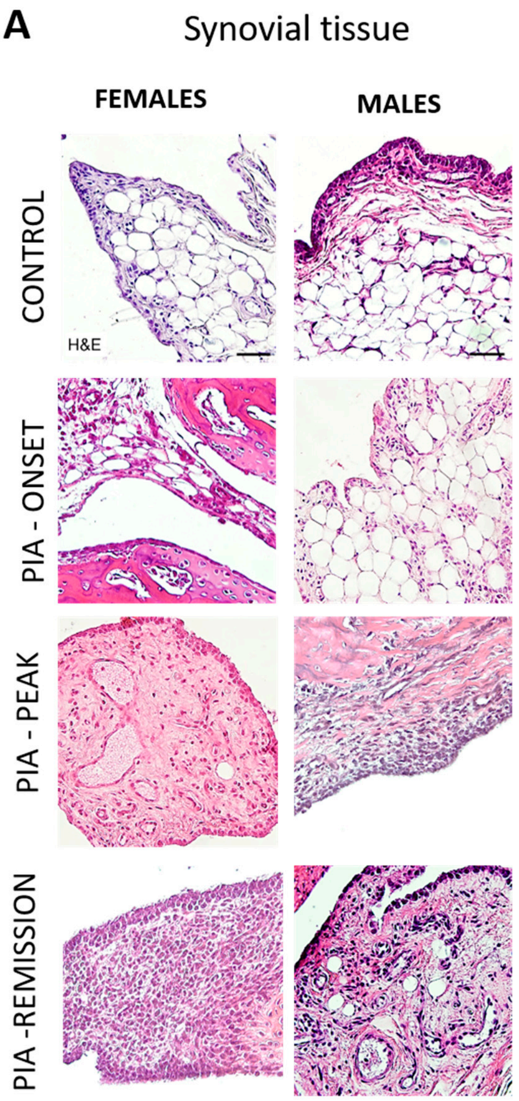

B

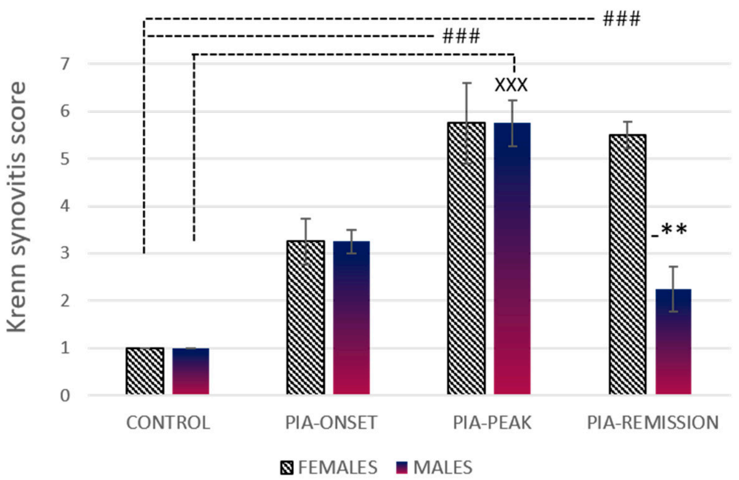

C

Variable	Gender	Clinical score
Krenn synovitis score	Males	0.844***
	Females	0.811***

Figure S1. Pristane-induced arthritis causes greater inflammation in synovial tissue in female than in male rats. (A) Representative photomicrographs show staining with H&E for measuring the level of inflammation (Krenn scoring system) in paraffin-embedded sections of the metatarsophalangeal joints obtained from male and female DA rats: control (treated with saline); PIA onset (between 9th and 12th day after induction); PIA peak (between 16th and 20th day after induction); PIA remission (between 20th and 25th day after induction). Scale bars indicate 50 μm. Some photomicrographs of the Figure S1A are adapted from Omrčen, et al. 2021 [8]. (B) Krenn synovitis score of metatarsophalangeal joints. Synovitis was scored by two independent observers under light microscope (3 slides/rat × 6 rats/group = 18 slides/group and total 24 rats). Values are expressed as mean value ± SD. One-way ANOVA followed by the post hoc Scheffé test: * difference between male and female rats; ^{X} difference between male control and male PIA rats; # difference between female control and female PIA rats; ^{XXX} $p < 0.001$; ### $p < 0.001$; ** $p < 0.01$. (C) Pearson's correlation between Krenn synovitis score and clinical score: *** $p < 0.001$.

A

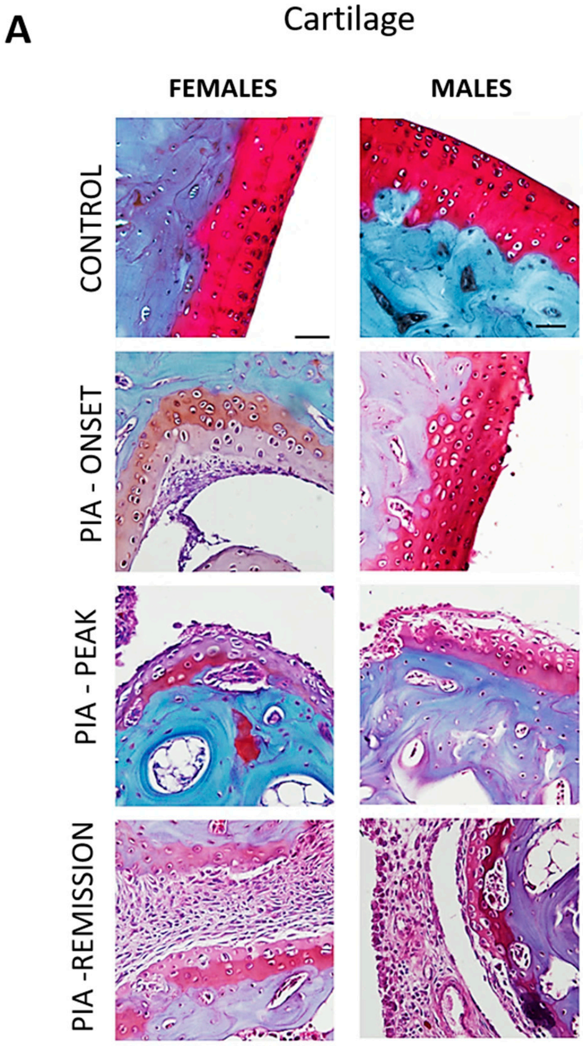

B

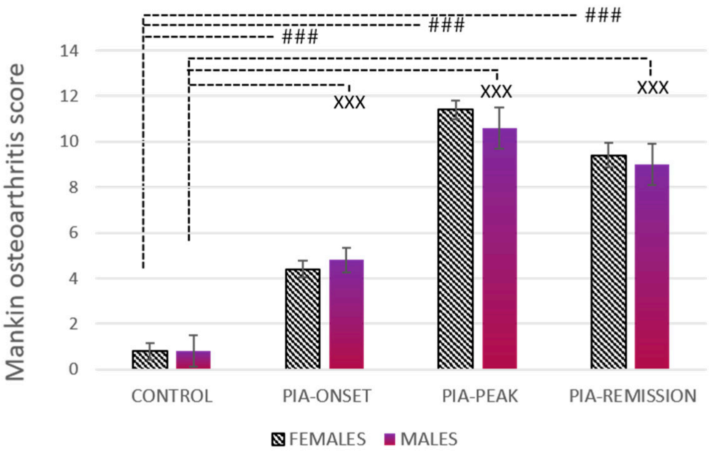

C

Variable	Gender	Clinical score
Mankin osteoarthritis score	Males<br>Females	0.884***<br>0.895***

Figure S2. Pristane-induced arthritis causes greater articular cartilage damage in females compared to males. (A) Photomicrographs show articular cartilage stained with histological Safranin O/Fast Green FCF staining to grade articular cartilage damage according to the modified Mankin scale on paraffin sections of the metatarsophalangeal joints obtained from male and female DA rats: control (treated with saline); PIA onset (between 9th and 12th day after induction); PIA peak (between 16th and 20th day after induction); PIA remission (between 20th and 25th day after induction). Scale bars indicate 50 μm. (B) Degree of cartilage damage of metatarsophalangeal joints according to the modified Mankin scale. The damage was scored by two independent observers under light microscope (3 slides/rat × 6 rats/group = 18 slides/group and total 24 rats). Values are expressed as mean value ± SD. One-way ANOVA followed by the post hoc Scheffé test: ^{X} difference between male control and male PIA rats; # difference between female control and female PIA rats; ^{XXX} $p < 0.001$; ### $p < 0.001$. (C) Pearson's correlation between Mankin osteoarthritis score and clinical score: *** $p < 0.001$.

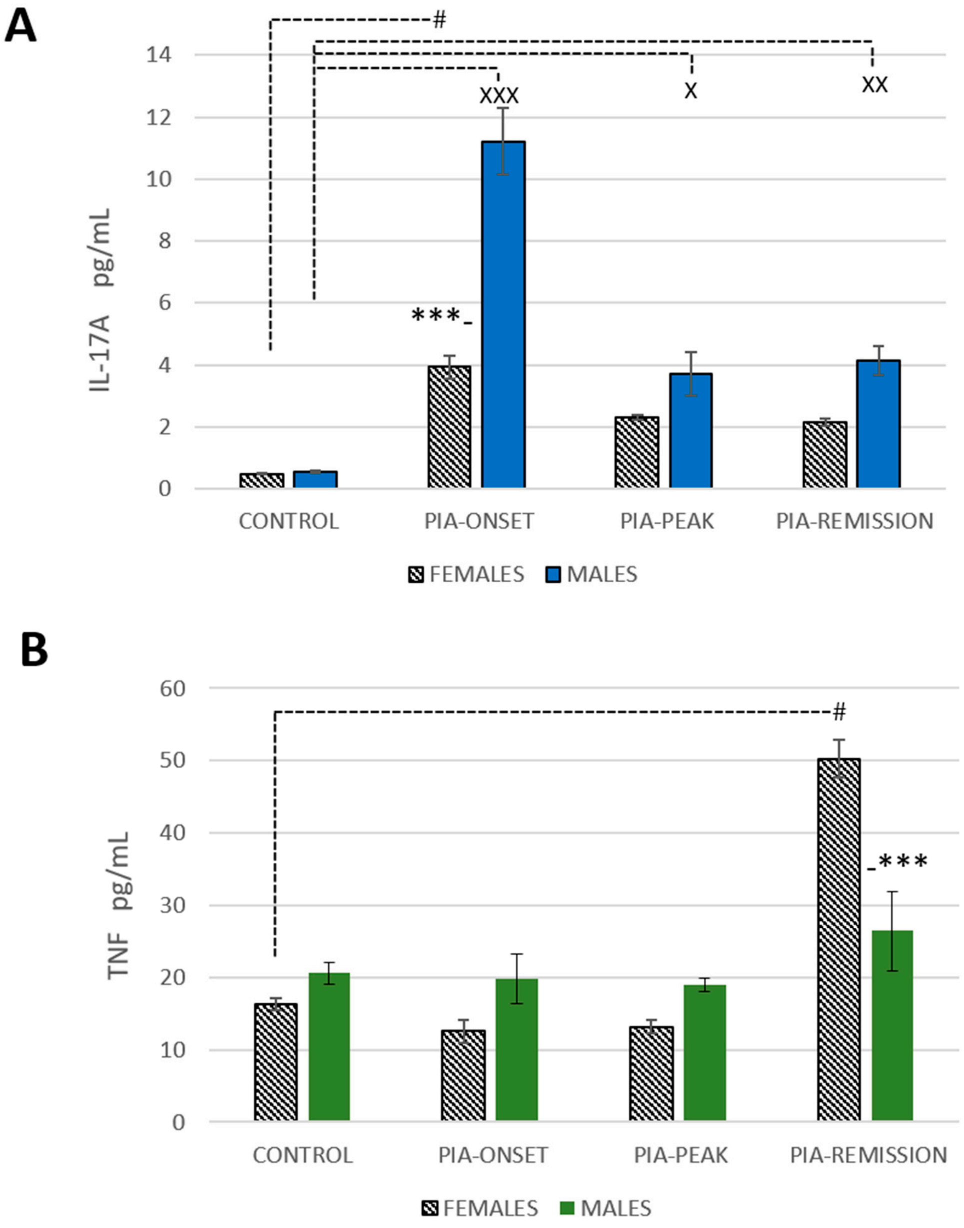

Figure S3. Expression profiles of TNF and IL-17A in serum during PIA. (A) Expression profile of TNF in serum. (B) Expression profile of IL-17A in serum. Both profiles are evaluated with ELISA in serum from male and female DA rats: control (treated with saline); PIA onset (between 9th and 12th day after induction); PIA peak (between 16th and 20th day after induction); PIA remission (between 20th and 25th day after induction). Values in pg/mL are expressed as mean ± SD (10 rats/group, N = 40). One-way ANOVA followed by the post hoc Scheffé test: * difference between male and female rats; ^{X} difference between male control and male PIA rats; # difference between female control and female PIA rats; # $p < 0.05$; ^{X} $p < 0.05$; ^{XX} $p < 0.01$; ^{XXX} $p < 0.001$, *** $p < 0.001$.
